# Editorial: NGS technologies of rare diseases diagnosis

**DOI:** 10.3389/fped.2022.1032359

**Published:** 2022-11-12

**Authors:** María L. Couce, Emiliano González-Vioque

**Affiliations:** ^1^ Diagnosis and Treatment Unit of Congenital Metabolic Diseases, Department of Paediatrics, University Clinical Hospital of Santiago de Compostela, IDIS, CIBERER, MetabERN, Santiago de Compostela, Spain; ^2^ Molecular Genetics Unit, Clinical Biochemistry Service, University Hospital Puerta de Hierro, Madrid, Spain

**Keywords:** DNA SEQUENCING, exome sequencing, genome sequencing, transcriptome, variants

**Editorial on the Research Topic** NGS technologies of rare diseases diagnosis By Couce ML, González-Vioque E. (2022) Front. Pediatr. 10: 1032359. doi: 10.3389/fped.2022.1032359

Of the estimated 7,000 rare diseases, 80% have a genetic cause (1). Because these diseases can be difficult to recognize based on clinical features alone, genetic testing is critical to help establish diagnosis (2). Genomic sequencing techniques allow the simultaneous analysis of hundreds to thousands of genes in a very short time and at low cost, and offer several strategies to facilitate the diagnosis of patients with suspected genetic disease, including gene panels and clinical exome sequencing (3), whole exome sequencing (WES) (4, 5), and whole genome sequencing (WGS) (6). However, it is becoming increasingly clear that an individual genome cannot be interpreted in isolation (7). WES and WGS data scaling necessitates automation of variant filtering to focus on clinically relevant genomic intervals and/or variants. On the other hand, analytical workflows for rare disease diagnosis involving processing of raw sequencing data, identification of pathogenic variants, and integration of clinical data to achieve robust genetic diagnosis are complex and not fully standardized (8, 9). Assigning pathogenicity to a rare variant identified using a bioinformatic algorithm and establishing a genetic diagnosis can be an extremely challenging process, and may require additional functional studies.

This Special Issue of *Frontiers in Genetics* “NGS Technologies of Rare Diseases Diagnosis” contains 33 studies about prenatal diagnostic technologies, phenotype expansions, added value of gene expression monitoring, and functional studies in the field of rare diseases, particularly those affecting the pediatric population. This collection of articles underscores the recent growth and extensive interest in research on this topic.

Prenatal research studies (Xu et al. (2021), 10) demonstrate the efficacy of cell-free DNA-based noninvasive prenatal screening (NIPS) diagnostic technologies for common trisomies in low-risk and twin pregnancies. However, the authors conclude that NIPS cannot replace invasive prenatal diagnostic techniques, and recommend prenatal diagnosis for fetuses with abnormal ultrasound findings.

Seven original studies have examined how WES can be used to identify genetic variants in rare diseases (Yang et al., Fareed et al., Zhang S et al., Huang et al., Giu et al., Zhang F et al., Yu et al.). The findings of these studies have expanded the variant spectrum of several rare diseases, revealing clearer relationships with clinical phenotypes, improving our understanding of the underlying etiology, and contributing to more rapid genetic diagnosis and subsequent genetic counseling. In their study, Yu et al. examined a fetus with structural brain abnormalities and identified a *de novo*, likely pathogenic variant of *TAOK1* (11). In addition, of the 19 case reports, 16, (Zhang J et al., Nabouli et al., Wang et al., Luo et al., Zhang P et al., Lin et al., Carneiro et al., Andreeva et al., Zhang T et al., Liu et al., Qiao et al., Ji et al., Zhou et al., Li et al., Tang et al., Hu et al.) describe the identification of a new variant, in known genetic diseases. Interestingly*,* Zaytseva et al. (2022) describe a loss-of-function variant in *ABCC9* that is associated with ventricular fibrillation. And, two of the case reports describe patients with 2 concomitant conditions Su et al. (2021), Kim et al. (2021) describe a case of a 7-year-old boy with two X-linked diseases, Duchenne muscular dystrophy (DMD) and frontometaphyseal dysplasia 1 (FMD1); while Shu et al. report a case of MEGDEL syndrome coinciding with SATB2-associated syndrome. Particularly noteworthy is the case report by Luo et al., which describes a novel frameshift mutation of *α-Actin 1* (ACTN1) in a Chinese family with macrothrombocytopenia and mild bleeding (NM_001130004: c.398_399insTGCG, p.F134AfsX60). This variant was identified in the proband and his mother, but was absent in other unaffected family members. Western blot revealed that expression of *α*-actin 1 in the proband was decreased markedly indicating that the novel frameshift mutation may induce non-sense-mediated mRNA decay. These findings not only broaden the spectrum of *ACTN1* variants, but also confirmed diagnosis of inherited macrothrombocytopenia, which may facilitate the management and prognosis of the members of the family in question.

The opinion article by Woo et al. (2021) discusses the inclusion of *GBA1* in many NGS analyses for Parkinson's disease, and the importance of considering the effects of the nearby homologous pseudogene. Recombinant alleles in *GBA1* identified in Gaucher disease and Parkinson's disease patients could be missed by relying on NGS analysis alone without Sanger sequencing validation.

Gene expression monitoring was also addressed in this special issue. Villate et al. (12) contribute a brief report on the importance of performing RNA functional assays in order to determine the clinical significance of intronic variants, and to facilitate genetic counseling and clinical management of patients and their relatives. The authors undertook clinical characterization of a novel splice variant in *NSD1* that causes familial Sotos syndrome. Their findings help highlight the importance of using *in silico* prediction tools to detect potential alterations in the splicing process. RNA-seq has emerged as a useful tool in the field of rare diseases, enabling the identification of new disease mechanisms and helping us to better understand the information generated by DNA sequencing.

And finally, functional analysis were included in three original studies (13, 14). Chen et al. (2021) characterized gene variants in Han Chinese patients with hypospadias, identifying 1 *de novo* missense variant loci in *AR gene,* and conducted *in vivo* and *in vitro* functional studies that provide molecular evidence that the consequent p.I817N amino acid change may significantly reduce AR transcriptional function, leading to hypospadias. Shen et al. are the first to describe a *B3GALT6*-dominant variant leading to Ehlers–Danlos disease, and their functional experiments confirm that the R295C variant plays a loss-of-function role, while the elongated variant (p.L170fs*268) may exert a dominant-negative effect. Votsi et al. studied a novel *SPG7* pathogenic variant in a Cypriot family with autosomal recessive spastic ataxia, and performed functional studies showing that the variant does not affect RNA or protein expression or protein localization. However, their findings reveal aberrant mitochondrial morphology, suggesting mitochondrial dysfunction and further demonstrating the pathogenicity of the identified variant.

## Conclusion

This special issue provides a useful summary of progress made in the field of NGS technologies applied to genetic medicine, particularly in the area of rare diseases. These approaches have helped improve diagnostic capabilities as well as expanding our knowledge of the molecular basis of these diseases, with important clinical and public health implications. Nonetheless, clinical studies with longer follow-up periods will be necessary to establish recommendations to ensure adequate and earlier diagnosis.
